# Relationship between inflammatory markers and long-term prognosis in ICU patients with acute non-ST-segment elevation myocardial infarction

**DOI:** 10.3389/fcvm.2025.1577385

**Published:** 2025-08-21

**Authors:** Yanze Li, Hongjin Jin, Guolin Zhang, Yangyou Zhang, Yanchun Ding

**Affiliations:** Departments of Cardiology, The Second Hospital of Dalian Medical University, Dalian, Liaoning, China

**Keywords:** inflammatory markers, inflammatory indicator, NSTEMI, ICU, predictive model, MIMIC-IV, NLR, NLPR

## Abstract

**Objective:**

This study aims to investigate the relation of inflammatory markers to the long-term prognosis of patients with severe non-ST-segment elevation myocardial infarction (NSTEMI) in the intensive care unit (ICU), and to further develop a predictive model for their long-term outcomes.

**Methods:**

This study utilized data on eligible NSTEMI patients from the Medical Information Mart for Intensive Care IV (MIMIC-IV) database. Patients were grouped based on mortality outcomes. The link of inflammatory markers to all-cause mortality (ACM) at 180 and 360 days in the ICU was analyzed through the Cox proportional hazards model and restricted cubic spline (RCS) curves. Survival differences across groups were evaluated via Kaplan–Meier (KM) survival analysis. The sample population was randomized into training and validation sets, and a novel prediction model for the risk of long-term death in ICU-admitted NSTEMI patients was constructed in the training group and validated in both groups.

**Results:**

1,607 NSTEMI patients were encompassed, with ACM rates of 9.7% at 180 days and 12.9% at 360 days. Multivariable Cox proportional hazards model analysis revealed that, in contrast to the low-level group (Q1), higher levels of neutrophil-to-lymphocyte ratio(NLR), neutrophil-to-lymphocyte-platelet ratio (NLPR), red blood cell distribution width (RDW), systemic immune-inflammation index (SII), and systemic inflammation response index (SIRI) were positively associated with ACM within 180 days and 360 days (all *P* < 0.05). The novel predictive model demonstrated high prognostic accuracy for long-term death in ICU-admitted NSTEMI individuals, with areas under the receiver operating characteristic (ROC) curve (AUC) of 0.730 in the training set and 0.751 in the validation set. Calibration curves revealed good concordance between predicted and observed probabilities.

**Conclusion:**

NLR, NLPR, and RDW are independent risk factors for long-term death in the ICU-admitted NSTEMI population. The long-term prognostic prediction model constructed for NSTEMI patients based on the aforementioned associations demonstrates high clinical predictive value.

## Introduction

Owing to advances in the research on coronary heart disease (CHD) and improvements in preventive and therapeutic measures, the global incidence of CHD tends to decline (1). However, in multiple low- and middle-income countries, its incidence continues to rise annually. In China, as of 2023, there were approximately 11.39 million CHD patients, and CHD has become one of the leading causes of mortality among middle-aged and old populations (2), imposing a substantial healthcare burden and economic strain. Among the clinical manifestations of CHD, acute myocardial infarction (AMI) represents the most serious form. The incidence and death of AMI have been steadily increasing due to population aging and changes in contemporary lifestyles. Among AMI types, acute non-ST-segment elevation myocardial infarction (NSTEMI) affects a larger population. Furthermore, a growing number of severe cases are admitted to intensive care units (ICUs) for close monitoring and treatment. ICU patients with NSTEMI often present with complex conditions, and multiple factors influence their prognosis. Therefore, risk prediction and management of long-term outcomes in severe NSTEMI patients have become increasingly critical.

Extensive research into the pathophysiology of CHD has identified oxidative stress and inflammatory processes as key contributors to the progression of CHD. In particular, inflammation has a key role throughout the entire course of CHD. In the early stages of CHD, the innate immune system, including monocyte-macrophage cells, participates in phagocytosis of low-density lipoprotein cholesterol (LDL-C) in the subendothelial space and boosts its oxidative modification and transformation into foam cells (3), thereby promoting the formation of lipid plaques. Simultaneously, macrophages and vascular endothelial cells release pro-inflammatory mediators like interleukin (IL)-6, C-reactive protein (CRP), as well as other inflammatory substances, which further exacerbate disease progression through pro-inflammatory, pro-coagulatory, and smooth muscle cell proliferative pathways (4). Therefore, different inflammatory cells and mediators not only reflect the overall status of CHD but also hold the potential for prognostic prediction.

To date, many studies have investigated the association between commonly used hematologic indices and coronary artery disease (CAD). Among these, composite inflammatory markers, such as the neutrophil-to-lymphocyte ratio (NLR), platelet-to-lymphocyte ratio (PLR), neutrophil-to-lymphocyte-platelet ratio (NLPR), systemic inflammation response index (SIRI), aggregate index of systemic inflammation (AISI) and systemic immune-inflammation index (SII), have garnered increasing attention for their integration of multiple hematologic parameters, thereby enhancing their utility in cardiovascular risk assessment. However, previous research has not thoroughly elucidated the prognostic value of these inflammatory indices across different subtypes of CAD. Given the higher clinical prevalence of NSTEMI, further investigation into risk stratification and management in this patient population is of particular importance.

Therefore, the main objectives of our study are to elucidate the correlations of various composite inflammatory indices (including NLR, PLR, SII, SIRI, AISI, NLPR, and RDW) with long-term prognosis in severe NSTEMI individuals in the ICU, identify significant markers, develop a novel prognostic prediction model, and evaluate its predictive performance.

## Materials and methods

### Data source

The sample population and data for our study were derived from the Medical Information Mart for Intensive Care IV (MIMIC-IV, version 2.2), a critical care database encompassing 2008–2019 records. This database, established collaboratively by the Laboratory for Computational Physiology at the Massachusetts Institute of Technology (MIT), Beth Israel Deaconess Medical Center (BIDMC), and Philips Healthcare, includes data of all medical and surgical ICU patients at BIDMC. All MIMIC-IV patient information is anonymized and publicly accessible. This database has been reviewed and approved by the Institutional Review Boards of MIT and BIDMC for scientific research purposes. The principal investigator of this study, Yanze Li, has completed the requisite training on the National Institutes of Health (NIH) website and gained access to the MIMIC-IV and relevant certificate (Record ID: 60349904).

### Study population

**Figure 1:**
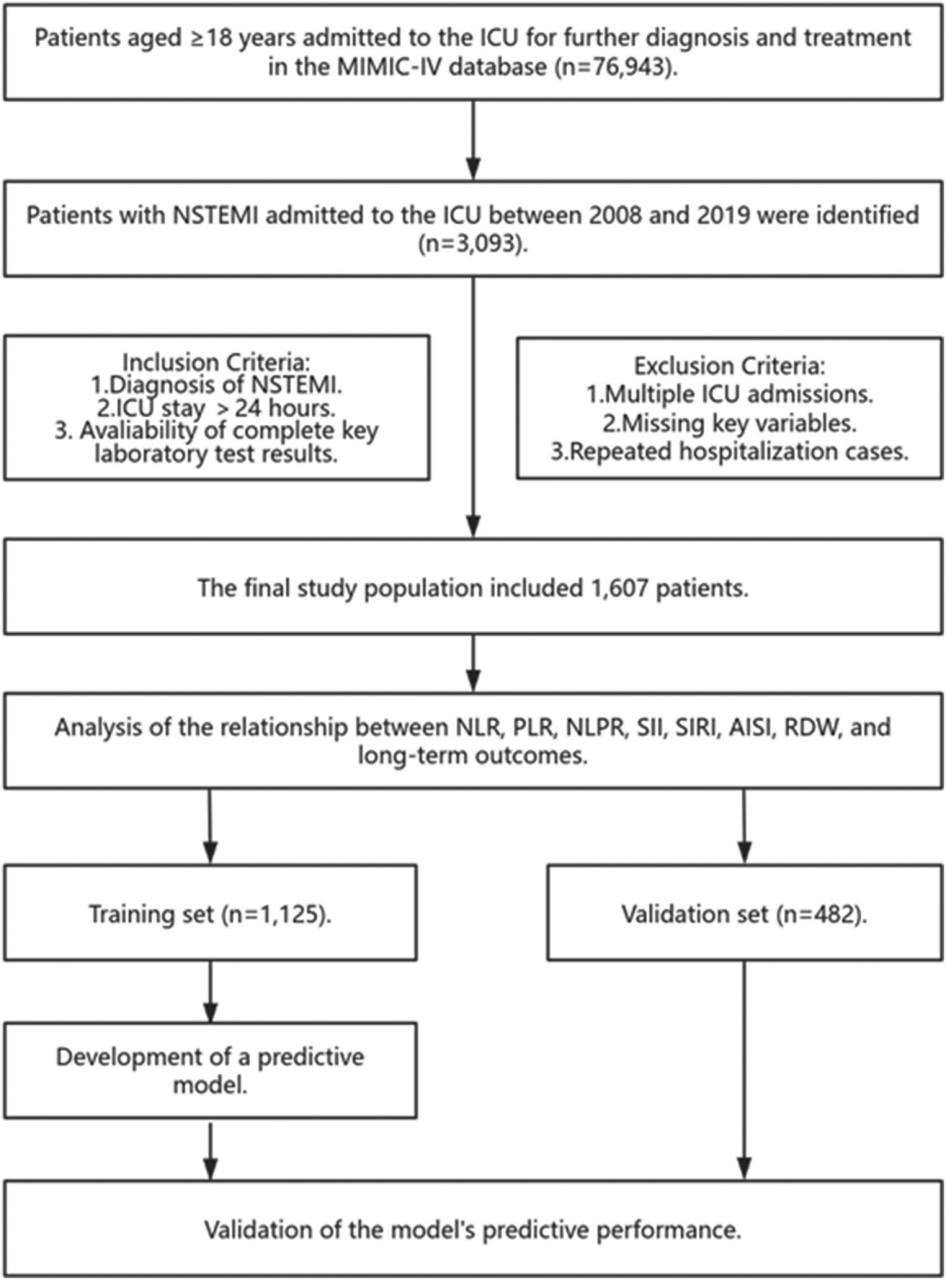
Research flowchart. MIMIC-IV, medical information mart for intensive care IV; ICU, intensive care unit; NSTEMI, non-ST segment elevation myocardial infarction; NLR, neutrophil-to-lymphocyte ratio; PLR, platelet-to-lymphocyte ratio; NLPR, neutrophil-to-lymphocyte-to-platelet ratio; SII, systemic immune-inflammation index; SIRI, systemic inflammation response index; AISI, aggregate index of systemic inflammation; RDW, red cell distribution width.

The study population included 3,093 patients diagnosed with NSTEMI in the MIMIC database as per the International Classification of Diseases, 10th Revision (ICD-10) code I21.4. Our exclusion criteria were: (1). repeated hospitalizations or multiple ICU admissions; (2). an ICU stay shorter than 24h; (3). missing critical laboratory results like neutrophil count (NEUT), lymphocyte count (LC), platelet count (PLT), monocyte count (MONO), and RDW (Figure 1).

### Data extraction

The following data were extracted via PostgreSQL 16: demographic characteristics [sex, age, race, body mass index (BMI)], vital signs [blood pressure, heart rate (HR), respiratory rate (RR), oxygen saturation, temperature], first laboratory test results upon ICU admission [hemoglobin, red blood cell count (RBC), NEUT, LC, PT, RDW], comorbidities, medications, other treatments, and scoring systems (Sequential Organ Failure Assessment(SOFA), Simplified Acute Physiology Score II(SAPS II), Glasgow Coma Scale (GCS)) from the MIMIC database. The primary endpoint was all-cause mortality (ACM) in 360 days of ICU entry. The secondary endpoint was ACM within 180 days of ICU admission. Data preprocessing was performed through R 4.4.1. Variables with over 20% missing values (excluding key variables: NEUT, LC, PT, RDW) were removed. Missing values for variables with less than 20% missing data were imputed through multiple imputations. Outliers in the data were addressed by replacing values above the 99th percentile with the 99th percentile value and values below the 1st percentile with the 1st percentile value.

### Definition of inflammatory markers

NLR is defined as the ratio of NEUT to LC. PLR represents the ratio of PLT to LC. NLPR is defined as the ratio of NEUT to the product of LC and PLT. AISI represents the product of NEUT, MONO, and PLT divided by LC. SII represents the product of PLT and NEUT divided by LC. SIRI is defined as the product of NEUT and MONO divided by LC.

### Statistical analysis

The Shapiro–Wilk test was performed to determine the normality of continuous variables. Normally distributed variables were shown in mean ± standard deviation (SD) and compared between groups through the *t*-test. Non-normally distributed ones were reported as median and interquartile range (IQR) and compared via the Mann–Whitney *U*-test. Categorical variables were expressed as frequencies and percentages, with comparisons across groups performed via the chi-squared or Fisher's exact test.

Each inflammatory marker was categorized into four levels based on its respective quartile cut-off values. Kaplan–Meier (KM) survival analysis was then performed to evaluate differences in 180-day and 360-day survival probabilities across these levels. Restricted cubic spline (RCS) analysis was further conducted to investigate potential nonlinear relationships between each inflammatory marker and the occurrence of endpoint events.

To further evaluate the independent associations between different inflammatory markers and outcome variables, multivariable Cox proportional hazards regression analysis was carried out through three models to adjust for various covariates. Model 1 was a crude model with no confounding factors. Model 2 was adjusted for sex, age, race, and BMI as covariates. Subsequently, the variance inflation factor (VIF) was calculated for each covariate to test for multicollinearity, and variables with VIF > 5 were excluded to ensure relative independence among the covariates. Model 3 was additionally adjusted for vital signs [systolic blood pressure (SBP), diastolic blood pressure (DBP), HR, RR, oxygen saturation, body temperature], laboratory parameters (blood urea nitrogen(BUN), serum creatinine (SCr), blood glucose, hemoglobin, mean corpuscular hemoglobin, serum sodium, serum potassium), comorbidities (Afib, acute kidney injury(AKI), acute respiratory failure (ARF), chronic heart failure (CHF), diabetes, dyslipidemia), medications and treatments (angiotensin-converting enzyme inhibitors, angiotensin II receptor blockers, beta-blockers, aspirin, statins, non-vitamin K antagonist oral anticoagulants, invasive mechanical ventilation), and clinical scoring systems (SAPS II, SOFA).

Our findings were further integrated with commonly used prognostic scoring systems to develop a new model for forecasting long-term prognosis in NSTEMI sufferers in the ICU. The study population was randomized into a training group and a validation group in a 7:3 ratio. In the former, the least absolute shrinkage and selection operator (LASSO) regression was applied to note predictive variables with the greatest impact on the primary endpoint, aiming to enhance model accuracy and generalizability. The selected variables were incorporated into logistic regression, and a nomogram was constructed. Finally, the forecasting performance of the novel model was assessed in both groups. The area under the curve (AUC) and calibration curve were used to assess the performance of the model. All statistical analyses were enabled by R 4.4.1. A two-sided test was employed for every analysis and *P* < 0.05 suggested statistical significance.

## Results

### Baseline characteristics

This study ultimately enrolled 1,607 NSTEMI patients in the ICU. The median (IQR) age of the cohort was 70 (61.0; 78.0) years, and 62.2% (*N* = 999) of participants were male. Among the patients, 1,400 (87.1%) survived for more than 360 days. Their baseline characteristics are presented in Table 1. Compared to the survival cohort, the non-survival cohort exhibited notably higher age [median: 76.0, IQR [67.5; 85.0] vs. median: 69.0, IQR [60.0; 77.0], *P* < 0.001] and consisted of more White patients (83.6% vs. 16.4%, *P* = 0.009). Furthermore, the non-survival group had lower BMI [median: 26.7, IQR [23.4; 30.9] vs. median: 28.4, IQR [24.8; 32.9], *P* = 0.001] and a risen incidence of ARF (43.5% vs. 30.0%, *P* < 0.001) but a decreased prevalence of dyslipidemia (59.9% vs. 81.3%, *P* < 0.001). Notably, the non-survival group had higher SOFA 24-hour scores [median: 2.00, IQR [1.00; 4.00] vs. median: 1.00, IQR [0.00; 4.00], *P* = 0.003] and SAPS II scores (median: 45.0, IQR [37.0; 52.0] vs. median: 36.0, IQR [29.0; 44.0], *P* < 0.001). Additionally, the non-survival group exhibited higher NEUT and RDW, along with lower PLT and LC. Nevertheless, statistically significant differences were not noted across groups in other comorbidities, including Afib, AKI, and CHF.

**Table 1:** Baseline characteristics of ICU patients with NSTEMI.

Characterisitics	All	Survival	Death	*P*-value
	*N* = 1,607	*N* = 1,400	*N* = 207	
Age, years	70.0 (61.0;78.0)	69.0 (60.0;77.0)	76.0 (67.5;85.0)	<0.001
BMI, kg/m^2^	28.2 (24.5;32.8)	28.4 (24.8;32.9)	26.7 (23.4;30.9)	0.001
Race, n (%)				0.009
White	1,223 (76.1%)	1,050 (75.0%)	173 (83.6%)	
Non white	384 (23.9%)	350 (25.0%)	34 (16.4%)	
Gender, n (%)				0.086
Male	999 (62.2%)	882 (63.0%)	117 (56.5%)	
Female	608 (37.8%)	518 (37.0%)	90 (43.5%)	
Vital sign				
SBP, mmHg	130 (118;143)	130 (119;143)	130 (115;140)	0.068
DBP, mmHg	71.0 (62.0;80.0)	72.0 (62.0;80.0)	69.0 (59.5;78.0)	0.001
Heart rate, bpm	83.0 (74.0;96.0)	82.0 (73.0;94.0)	91.0 (78.0;105)	<0.001
Respiratory rate, rpm	18.0 (14.0;22.0)	18.0 (14.0;22.0)	20.0 (16.0;24.5)	<0.001
SpO2, %	98.0 (95.0;100)	98.0 (96.0;100)	97.0 (94.0;100)	<0.001
Temperature, °F	98.1 (97.7;98.6)	98.1 (97.7;98.6)	98.2 (97.7;98.6)	0.983
Laboratory				
BUN, mg/dl	21.0 (15.0;31.0)	20.0 (15.0;30.0)	26.0 (18.0;48.0)	<0.001
Creatinine, mg/dl	1.10 (0.90;1.50)	1.10 (0.80;1.40)	1.30 (1.00;2.00)	<0.001
Glucose, mg/dl	124 (100;177)	122 (99.0;174)	142 (104;188)	0.005
Hemoglobin, g/dl	12.4 (10.9;13.9)	12.6 (11.2;14.0)	11.0 (9.10;12.8)	<0.001
Lymphocyte, K/ul	1.42 (0.90;2.03)	1.50 (0.95;2.06)	1.09 (0.67;1.68)	<0.001
MCH, fl	30.2 (28.8;31.6)	30.2 (28.9;31.6)	30.0 (28.2;31.6)	0.207
Monocytes, K/ul	0.66 (0.48;0.89)	0.66 (0.48;0.88)	0.66 (0.46;0.95)	0.908
Neutrophils, K/ul	6.68 (4.60;10.1)	6.48 (4.49;9.87)	7.98 (5.64;11.7)	<0.001
Platelet, K/ul	219 (176;275)	222 (180;275)	198 (150;274)	0.001
RDW, %	13.8 (13.1;14.8)	13.8 (13.1;14.7)	14.6 (13.6;16.5)	<0.001
Potassium,mEq/L	4.20 (3.90;4.70)	4.20 (3.90;4.60)	4.40 (3.90;5.00)	0.007
Sodium, mEq/L	139 (136;141)	139 (137;141)	138 (135;141)	0.001
Comorbidities, *n* (%)				
AF	751 (46.7%)	648 (46.3%)	103 (49.8%)	0.39
AKF	994 (61.9%)	854 (61.0%)	140 (67.6%)	0.079
ARF	510 (31.7%)	420 (30.0%)	90 (43.5%)	<0.001
CHF	1,005 (62.5%)	873 (62.4%)	132 (63.8%)	0.753
Diabetes	876 (54.5%)	777 (55.5%)	99 (47.8%)	0.046
Dyslipidemia	1,262 (78.5%)	1,138 (81.3%)	124 (59.9%)	<0.001
Medication and Interventions, *n* (%)				
ACEI	819 (51.0%)	760 (54.3%)	59 (28.5%)	<0.001
ARB	349 (21.7%)	328 (23.4%)	21 (10.1%)	<0.001
Aspirin	1,511 (94.0%)	1,336 (95.4%)	175 (84.5%)	<0.001
Beta blocker	1,489 (92.7%)	1,329 (94.9%)	160 (77.3%)	<0.001
Statin	1,483 (92.3%)	1,318 (94.1%)	165 (79.7%)	<0.001
Xaban	259 (16.1%)	232 (16.6%)	27 (13.0%)	0.235
IMV	1,045 (65.0%)	921 (65.8%)	124 (59.9%)	0.114
Score				
SAPSII	37.0 (30.0;46.0)	36.0 (29.0;44.0)	45.0 (37.0;52.0)	<0.001
SOFA 24 h	2.00 (0.00;4.00)	1.00 (0.00;4.00)	2.00 (1.00;4.00)	0.003
Inflammation indicators				
SII	1,020 (563;1,961)	968 (550;1,867)	1,444 (766;2,790)	<0.001
SIRI	2.89 (1.57;6.24)	2.73 (1.53;5.72)	4.75 (2.40;8.69)	<0.001
NLR	4.71 (2.76;8.71)	4.38 (2.60;7.97)	7.46 (4.21;12.5)	<0.001
PLR	155 (101;245)	153 (101;241)	186 (107;282)	0.021
NLPR	0.02 (0.01;0.04)	0.02 (0.01;0.04)	0.04 (0.02;0.08)	<0.001
AISI	640 (314;1,393)	601 (310;1,302)	961 (346;1,812)	<0.001

ACEI, angiotensin-converting enzyme inhibitors; AF, atrial fibrillation; AISI, aggregate index of systemic inflammation; AKF, acute kidney failure; ARB, angiotensin receptor blocker; ARF, acute respiratory failure; BMI, body mass index; BUN, blood urea nitrogen; CHF, congestive heart failure; DBP, diastolic blood pressure; IMV, invasive mechanical ventilation; MCH, mean corpsular hemoglobin; MCV, mean corpuscular volume; NLPR, neutrophil to lymphocyte platelet ratio; NLR, neutrophil-lymphocyte ratio; PLR, platelet-lymphocyte ratio; RDW, red blood cell volume distribution width; SAPSII, simplified acute physiology score II; SBP, diastolic blood pressure; SII, systemic inflammatory index; SIRI, systemic inflammatory response index; SOFA, sequential organ failure assessment; SpO2, saturation of peripheral oxygen.

### Associations between inflammatory markers and 180-day ACM in ICU patients with NSTEMI

**Figure 2:**
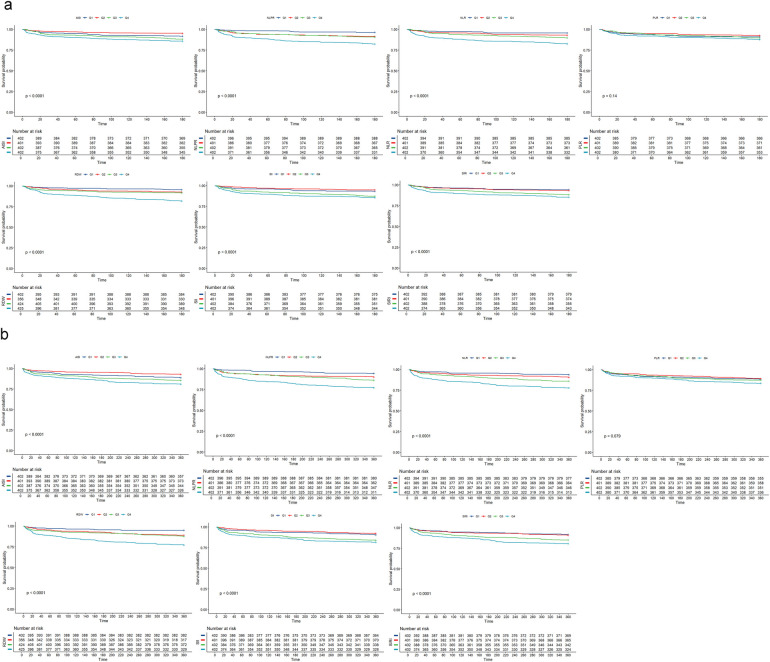
Kaplan–meier survival analysis of NSTEMI patients in the ICU. **(a)** K-M survival analysis of 180-day mortality in NSTEMI patients in ICU; **(b)** K-M survival analysis of 360-day mortality in NSTEMI patients in ICU; NLR, neutrophil-lymphocyte ratio; PLR, platelet-lymphocyte ratio; NLPR, neutrophil-lymphocyte platelet ratio; SII, systemic inflammatory index; SIRI, systemic inflammatory response index; AISI, inflammatory response integrated index; RDW, red blood cell distribution width.

KM survival analysis demonstrated notable differences in 180-day survival rates across varied levels of inflammatory markers (Figure 2a). Compared to the low-level subgroup (Q1), patients with high levels (Q4) of all inflammatory markers (NLR, NLPR, SII, SIRI, AISI, RDW), except PLR, exhibited the lowest survival rates, with statistically significant differences (*P* < 0.05).

After adjustments for baseline characteristics, vital signs, laboratory findings, comorbidities, medications, other treatments, and scoring systems, the multivariate Cox proportional hazards regression analysis (Table 2) revealed a positive association between elevated levels of NLR, NLPR, RDW, SII, and SIRI and 180-day ACM in ICU patients with NSTEMI. Therefore, increased levels of these markers can be considered independent risk factors for 180-day death in the patient population. Higher levels of NLR (Q1 (reference group), Q2 (HR = 1.567, 95% CI: 0.846–2.901, *P* = 0.153), Q3 (HR = 1.810, 95% CI: 0.997–3.284, *P* = 0.051), and Q4 (HR = 2.565, 95% CI: 1.458–4.510, *P* < 0.05), NLPR(Q1 (reference group), Q2 (HR = 1.820, 95% CI: 0.968–3.421, *P* = 0.063), Q3 (HR = 1.674, 95% CI: 0.890–3.148, *P* = 0.11), and Q4 (HR = 2.361, 95% CI: 1.289–4.323, *P* < 0.05), and RDW (Q1 (reference group), Q2 (HR = 1.289, 95% CI: 0.686–2.423, *P* = 0.430), Q3 (HR = 1.505, 95% CI: 0.823–2.753, *P* = 0.185), and Q4 (HR = 2.167, 95% CI: 1.190–3.946, *P* < 0.05) were linked to significantly elevated death risk in ICU patients with NSTEMI within 180 days.

**Table 2:** The association of each inflammatory indicator with 180-day ICU mortality in ICU patients with NSTEMI.

Categoris	Model1		Model2		Model3	
	HR (95% CI)	*P*-value	HR (95% CI)	*P*-value	HR (95% CI)	*P*-value
NLR ((Quartile)						
Q1	—		—		—	
Q2	1.675 (0.917, 3.060)	0.094	1.547 (0.845, 2.832)	0.157	1.567 (0.846, 2.901)	0.153
Q3	2.464 (1.400, 4.336)	0.002	2.127 (1.203, 3.761)	0.009	1.810 (0.997, 3.284)	0.051
Q4	4.445 (2.616, 7.551)	<0.001	3.812 (2.230, 6.516)	<0.001	2.565 (1.458, 4.510)	0.001
PLR (Quartile)						
Q1	—		—		—	
Q2	0.825 (0.508, 1.339)	0.435	0.817 (0.503, 1.328)	0.415	0.886 (0.529, 1.483)	0.645
Q3	1.132 (0.724, 1.772)	0.587	1.090 (0.696, 1.708)	0.707	1.203 (0.749, 1.933)	0.445
Q4	1.381 (0.898, 2.123)	0.141	1.219 (0.790, 1.881)	0.370	1.068 (0.668, 1.707)	0.783
RDW (Quartile)						
Q1	—		—		—	
Q2	1.656 (0.908, 3.021)	0.100	1.468 (0.803, 2.684)	0.213	1.289 (0.686, 2.423)	0.430
Q3	1.896 (1.074, 3.348)	0.027	1.663 (0.938, 2.948)	0.082	1.505 (0.823, 2.753)	0.185
Q4	4.349 (2.603, 7.265)	<0.001	4.212 (2.517, 7.049)	<0.001	2.167 (1.190, 3.946)	0.011
NLPR (Quartile)						
Q1	—		—		—	
Q2	2.507 (1.345, 4.671)	0.004	2.240 (1.198, 4.188)	0.012	1.820 (0.968, 3.421)	0.063
Q3	2.716 (1.469, 5.024)	0.001	2.231 (1.196, 4.161)	0.012	1.674 (0.890, 3.148)	0.110
Q4	5.501 (3.101, 9.758)	<0.001	4.635 (2.590, 8.296)	<0.001	2.361 (1.289, 4.323)	0.005
AISI (Quartile)						
Q1	—		—		—	
Q2	0.568 (0.323, 0.999)	0.050	0.543 (0.309, 0.956)	0.034	0.602 (0.337, 1.077)	0.087
Q3	1.442 (0.924, 2.251)	0.107	1.306 (0.834, 2.044)	0.243	1.367 (0.846, 2.207)	0.202
Q4	1.800 (1.172, 2.763)	0.007	1.605 (1.042, 2.471)	0.032	1.440 (0.917, 2.262)	0.113
SII (Quartile)						
Q1	—		—		—	
Q2	0.733 (0.411, 1.306)	0.292	0.690 (0.387, 1.232)	0.210	0.812 (0.446, 1.479)	0.496
Q3	1.935 (1.214, 3.085)	0.006	1.814 (1.136, 2.896)	0.013	1.999 (1.215, 3.291)	0.006
Q4	2.256 (1.429, 3.561)	<0.001	2.003 (1.265, 3.174)	0.003	1.746 (1.063, 2.869)	0.028
SIRI (Quartile)						
Q1	—		—		—	
Q2	1.182 (0.678, 2.061)	0.556	1.080 (0.618, 1.888)	0.787	1.004 (0.568, 1.772)	0.990
Q3	2.090 (1.269, 3.442)	0.004	1.831 (1.107, 3.030)	0.019	1.520 (0.897, 2.574)	0.120
Q4	2.708 (1.673, 4.384)	<0.001	2.260 (1.387, 3.682)	0.001	1.714 (1.033, 2.845)	0.037

**Figure 3:**
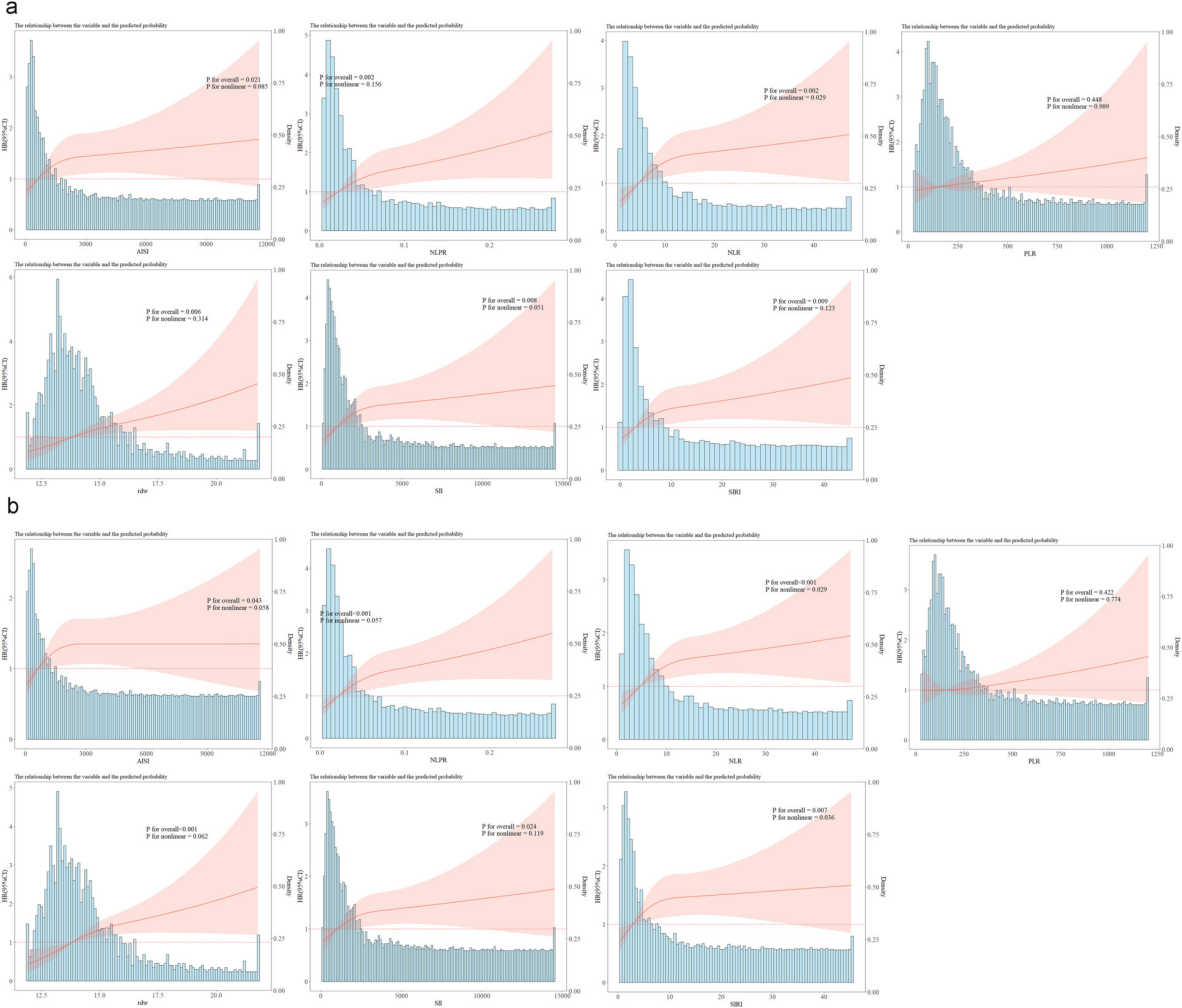
Restricted cubic spline analysis of inflammation indices (AISI, NLPR, NLR, PLR, RDW, SII, SIRI) and ICU mortality. **(a)** RCS analysis of each inflammatory indicator and 180-day mortality; **(b)** RCS analysis of each inflammatory indicator and 360-day mortality; NLR, neutrophil-lymphocyte ratio; PLR, platelet-lymphocyte ratio; NLPR, neutrophil-lymphocyte platelet ratio; SII, systemic inflammation index; SIRI, systemic inflammation response index; AISI, inflammatory response integrated index; RDW, red blood cell distribution width.

RCS analysis further demonstrated that, except for PLR, higher levels of all inflammatory markers, as continuous variables, were significantly linked to increased 180-day ACM (*P* < 0.05). A nonlinear positive relation of NLR to 180-day death was noted (P for nonlinearity = 0.029), as shown in Figure 3a.

### Association between inflammatory markers and 360-day ACM in NSTEMI patients in the ICU

KM survival analysis was also employed to investigate survival differences within the cohort over 360 days, as illustrated in Figure 2b. The results were consistent with those observed at 180 days: in contrast to patients in Q1, those in Q4, except for PLR, exhibited the lowest survival rates, with statistically significant differences.

The multivariate Cox regression analysis, further adjusted for baseline data, vital signs, laboratory tests, comorbidities, medication, and other treatments, as well as scoring variables, is presented in Table 3. In ICU patients with NSTEMI, ACM within 360 days was positively correlated with increased levels of NLR, NLPR, RDW, SII, and SIRI. Specifically, elevated NLR [Q1(reference group); Q2: HR = 1.325, 95% CI: 0.790–2.224, *P* = 0.286; Q3: HR = 1.621, 95% CI: 0.988–2.657, *P* < 0.056; Q4: HR = 2.205, 95% CI: 1.378–3.529, *P* < 0.05], NLPR [Q1(reference group); Q2: HR = 1.399, 95% CI: 0.824–2.274, *P* = 0.213; Q3: HR = 1.686, 95% CI: 1.013–2.807, *P* < 0.05; Q4: HR = 2.138, 95% CI: 1.306–3.501, *P* < 0.05], and RDW [Q1 (reference group); Q2: HR = 1.821, 95% CI: 1.039–3.191, *P* < 0.05; Q3: HR = 2.047, 95% CI: 1.188–3.528, *P* < 0.05; Q4: HR = 2.738, 95% CI: 1.587–4.726, *P* < 0.05] were associated with an elevated risk of mortality.

**Table 3:** The association of each inflammatory indicator with 360-day ICU mortality in ICU patients with NSTEMI.

Categoris	Model1		Model2		Model3	
	HR (95% CI)	*P*-value	HR (95% CI)	*P*-value	HR (95% CI)	*P*-value
NLR ((Quartile)						
Q1	—		—		—	
Q2	1.510 (0.909, 2.509)	0.111	1.414 (0.850, 2.352)	0.183	1.325 (0.790, 2.224)	0.286
Q3	2.320 (1.448, 3.717)	<0.001	2.032 (1.262, 3.271)	0.004	1.621 (0.988, 2.657)	0.056
Q4	3.935 (2.525, 6.132)	<0.001	3.434 (2.190, 5.384)	<0.001	2.205 (1.378, 3.529)	<0.001
PLR (Quartile)						
Q1	—		—		—	
Q2	0.905 (0.598, 1.369)	0.636	0.885 (0.585, 1.339)	0.563	0.925 (0.599, 1.431)	0.727
Q3	1.084 (0.730, 1.612)	0.689	1.027 (0.690, 1.528)	0.896	1.088 (0.717, 1.651)	0.691
Q4	1.437 (0.989, 2.089)	0.057	1.257 (0.862, 1.832)	0.235	1.054 (0.705, 1.576)	0.797
RDW (Quartile)						
Q1	—		—		—	
Q2	2.255 (1.315, 3.866)	0.003	2.011 (1.170, 3.455)	0.011	1.821 (1.039, 3.191)	0.036
Q3	2.553 (1.524, 4.276)	<0.001	2.240 (1.333, 3.765)	0.002	2.047 (1.188, 3.528)	0.010
Q4	5.010 (3.094, 8.111)	<0.001	4.873 (3.005, 7.900)	<0.001	2.738 (1.587, 4.726)	<0.001
NLPR (Quartile)						
Q1	—		—		—	
Q2	1.838 (1.090, 3.099)	0.022	1.680 (0.993, 2.841)	0.053	1.399 (0.824, 2.374)	0.213
Q3	2.597 (1.584, 4.258)	<0.001	2.190 (1.325, 3.621)	0.002	1.686 (1.013, 2.807)	0.045
Q4	4.599 (2.887, 7.326)	<0.001	4.003 (2.491, 6.431)	<0.001	2.138 (1.306, 3.501)	0.003
AISI (Quartile)						
Q1	—		—		—	
Q2	0.611 (0.381, 0.979)	0.040	0.582 (0.363, 0.933)	0.025	0.612 (0.377, 0.992)	0.047
Q3	1.314 (0.890, 1.939)	0.170	1.187 (0.802, 1.757)	0.391	1.172 (0.774, 1.775)	0.454
Q4	1.781 (1.232, 2.576)	0.002	1.584 (1.092, 2.297)	0.015	1.373 (0.933, 2.021)	0.108
SII (Quartile)						
Q1	—		—		—	
Q2	0.807 (0.502, 1.296)	0.375	0.757 (0.471, 1.218)	0.252	0.806 (0.494, 1.316)	0.389
Q3	1.746 (1.169, 2.608)	0.007	1.642 (1.098, 2.456)	0.016	1.616 (1.057, 2.471)	0.027
Q4	2.074 (1.402, 3.067)	<0.001	1.843 (1.242, 2.734)	0.002	1.523 (0.999, 2.322)	0.050
SIRI (Quartile)						
Q1	—		—		—	
Q2	1.099 (0.685, 1.763)	0.695	1.012 (0.630, 1.627)	0.960	0.906 (0.560, 1.466)	0.688
Q3	1.880 (1.230, 2.876)	0.004	1.668 (1.086, 2.563)	0.019	1.345 (0.858, 2.107)	0.196
Q4	2.539 (1.690, 3.815)	<0.001	2.144 (1.419, 3.241)	<0.001	1.606 (1.048, 2.462)	0.030

Finally, the RCS analysis revealed that, except for PLR, all inflammatory markers, when treated as continuous variables, were statistically related to ACM within 360 days. Specifically, NLR (P for nonlinear = 0.029), and SIRI (P for nonlinear = 0.036) exhibited a nonlinear positive correlation with ACM within 360 days (Figure 3b).

### Sensitivity analysis

Given that inflammation-related conditions such as malignancies and rheumatic immune diseases possibly influence the systemic inflammatory status of patients and thereby potentially confound the findings of this study, a sensitivity analysis was performed to assess the robustness of our results. Specifically, individuals diagnosed with malignant tumors or rheumatic immune diseases were excluded from the study population, and the multivariate Cox proportional hazards models were re-estimated to evaluate the associations between inflammatory indices and ACM at 180 and 360 days. The results indicated that NLR, RDW, and NLPR remained significantly associated with 180-day mortality (all *P* < 0.05), with HRs elevated compared to those in the original model. In contrast, after adjusting for all confounders, the associations of SII and SIRI with 180-day ACM were no longer statistically significant (Table 4). Similarly, the associations of NLR, RDW, and NLPR with 360-day ACM remained robust. Notably, SII also retained statistical significance in relation to 360-day ACM. However, the predictive significance of SIRI completely disappeared after adjustment for all potential confounders. Detailed results are presented in Table 5.

**Table 4:** Sensitivity analysis of the relationship between inflammatory indicator with 180-day mortality in ICU patients with NSTEMI.

Categoris	Model1		Model2		Model3	
	HR (95% CI)	*P*-value	HR (95% CI)	*P*-value	HR (95% CI)	*P*-value
NLR ((Quartile)						
Q1	—		—		—	
Q2	1.398 (0.685, 2.854)	0.357	1.281 (0.626, 2.620)	0.498	1.498 (0.713, 3.150)	0.286
Q3	2.436 (1.275, 4.654)	0.007	2.008 (1.045, 3.858)	0.036	1.924 (0.975, 3.796)	0.059
Q4	4.960 (2.723, 9.034)	<0.001	4.041 (2.199, 7.424)	<0.001	2.852 (1.497, 5.432)	0.001
PLR (Quartile)						
Q1	—		—		—	
Q2	0.697 (0.402, 1.207)	0.198	0.666 (0.384, 1.155)	0.148	0.912 (0.503, 1.655)	0.762
Q3	1.037 (0.630, 1.707)	0.886	0.980 (0.594, 1.617)	0.938	1.111 (0.640, 1.928)	0.708
Q4	1.253 (0.777, 2.023)	0.355	1.043 (0.643, 1.691)	0.864	0.908 (0.535, 1.543)	0.722
RDW (Quartile)						
Q1	—		—		—	
Q2	1.929 (0.969, 3.839)	0.061	1.718 (0.861, 3.429)	0.125	1.764 (0.843, 3.692)	0.132
Q3	2.076 (1.043, 4.132)	0.038	1.784 (0.893, 3.567)	0.101	1.862 (0.891, 3.892)	0.098
Q4	4.716 (2.537, 8.765)	<0.001	4.446 (2.385, 8.289)	<0.001	2.843 (1.376, 5.876)	0.005
NLPR (Quartile)						
Q1	—		—		—	
Q2	3.612 (1.646, 7.925)	0.001	3.074 (1.396, 6.771)	0.005	3.082 (1.382, 6.872)	0.006
Q3	3.331 (1.508, 7.356)	0.003	2.591 (1.162, 5.777)	0.020	1.876 (0.831, 4.238)	0.130
Q4	8.140 (3.892, 17.02)	<0.001	6.402 (3.028, 13.53)	<0.001	3.177 (1.453, 6.947)	0.004
AISI (Quartile)						
Q1	—		—		—	
Q2	0.473 (0.244, 0.917)	0.027	0.449 (0.231, 0.870)	0.018	0.570 (0.285, 1.139)	0.112
Q3	1.345 (0.817, 2.216)	0.244	1.202 (0.727, 1.987)	0.473	1.300 (0.745, 2.270)	0.356
Q4	1.769 (1.100, 2.845)	0.019	1.480 (0.916, 2.392)	0.109	1.336 (0.796, 2.242)	0.272
SII (Quartile)						
Q1	—		—		—	
Q2	0.705 (0.363, 1.367)	0.300	0.677 (0.349, 1.314)	0.249	0.791 (0.393, 1.592)	0.511
Q3	1.846 (1.083, 3.145)	0.024	1.671 (0.979, 2.851)	0.060	2.027 (1.134, 3.625)	0.017
Q4	2.395 (1.434, 3.999)	<0.001	2.045 (1.219, 3.431)	0.007	1.733 (0.973, 3.087)	0.062
SIRI (Quartile)						
Q1	—		—		—	
Q2	0.847 (0.444, 1.617)	0.615	0.727 (0.379, 1.394)	0.337	0.788 (0.406, 1.530)	0.481
Q3	1.833 (1.061, 3.166)	0.030	1.495 (0.860, 2.601)	0.154	1.292 (0.709, 2.355)	0.403
Q4	2.571 (1.528, 4.324)	<0.001	1.987 (1.169, 3.379)	0.011	1.452 (0.823, 2.563)	0.198

**Table 5:** Sensitivity analysis of the relationship between inflammatory indicator with 360-day mortality in ICU patients with NSTEMI.

Categoris	Model1		Model2		Model3	
	HR (95% CI)	*P*-value	HR (95% CI)	*P*-value	HR (95% CI)	*P*-value
NLR ((Quartile)						
Q1	—		—		—	
Q2	1.404 (0.766, 2.573)	0.272	1.313 (0.715, 2.411)	0.381	1.486 (0.791, 2.790)	0.218
Q3	2.356 (1.353, 4.100)	0.002	1.985 (1.134, 3.474)	0.016	1.883 (1.053, 3.366)	0.033
Q4	4.786 (2.866, 7.990)	<0.001	3.998 (2.374, 6.732)	<0.001	2.882 (1.664, 4.992)	<0.001
PLR (Quartile)						
Q1	—		—		—	
Q2	0.793 (0.492, 1.278)	0.341	0.742 (0.460, 1.197)	0.222	0.959 (0.575, 1.599)	0.871
Q3	1.056 (0.675, 1.651)	0.812	0.966 (0.616, 1.514)	0.879	1.028 (0.632, 1.671)	0.912
Q4	1.456 (0.958, 2.212)	0.078	1.177 (0.771, 1.796)	0.450	1.013 (0.641, 1.600)	0.956
RDW (Quartile)						
Q1	—		—		—	
Q2	2.517 (1.332, 4.758)	0.004	2.254 (1.190, 4.270)	0.013	2.453 (1.248, 4.820)	0.009
Q3	3.094 (1.655, 5.785)	<0.001	2.662 (1.419, 4.995)	0.002	2.936 (1.506, 5.725)	0.002
Q4	5.503 (3.052, 9.922)	<0.001	5.210 (2.884, 9.414)	<0.001	3.587 (1.834, 7.014)	<0.001
NLPR (Quartile)						
Q1	—		—		—	
Q2	2.859 (1.477, 5.536)	0.002	2.495 (1.284, 4.848)	0.007	2.531 (1.291, 4.965)	0.007
Q3	3.455 (1.812, 6.586)	<0.001	2.796 (1.453, 5.378)	0.002	2.150 (1.105, 4.182)	0.024
Q4	7.168 (3.901, 13.17)	<0.001	5.872 (3.162, 10.90)	<0.001	3.277 (1.719, 6.249)	<0.001
AISI (Quartile)						
Q1	—		—		—	
Q2	0.410 (0.229, 0.735)	0.003	0.390 (0.217, 0.700)	0.002	0.492 (0.268, 0.901)	0.022
Q3	1.254 (0.818, 1.924)	0.299	1.130 (0.734, 1.738)	0.580	1.160 (0.724, 1.856)	0.537
Q4	1.685 (1.124, 2.526)	0.012	1.407 (0.934, 2.120)	0.102	1.288 (0.833, 1.990)	0.255
SII (Quartile)						
Q1	—		—		—	
Q2	0.810 (0.467, 1.406)	0.455	0.768 (0.442, 1.335)	0.350	0.864 (0.484, 1.541)	0.620
Q3	1.768 (1.109, 2.817)	0.017	1.600 (1.002, 2.553)	0.049	1.735 (1.050, 2.866)	0.032
Q4	2.397 (1.536, 3.741)	<0.001	2.036 (1.299, 3.193)	0.002	1.694 (1.033, 2.780)	0.037
SIRI (Quartile)						
Q1	—		—		—	
Q2	0.957 (0.552, 1.656)	0.874	0.835 (0.480, 1.452)	0.523	0.894 (0.509, 1.570)	0.697
Q3	1.818 (1.124, 2.940)	0.015	1.517 (0.932, 2.471)	0.094	1.322 (0.782, 2.235)	0.298
Q4	2.673 (1.696, 4.212)	<0.001	2.109 (1.325, 3.358)	0.002	1.609 (0.983, 2.636)	0.059

### Development of a new predictive model

**Figure 4:**
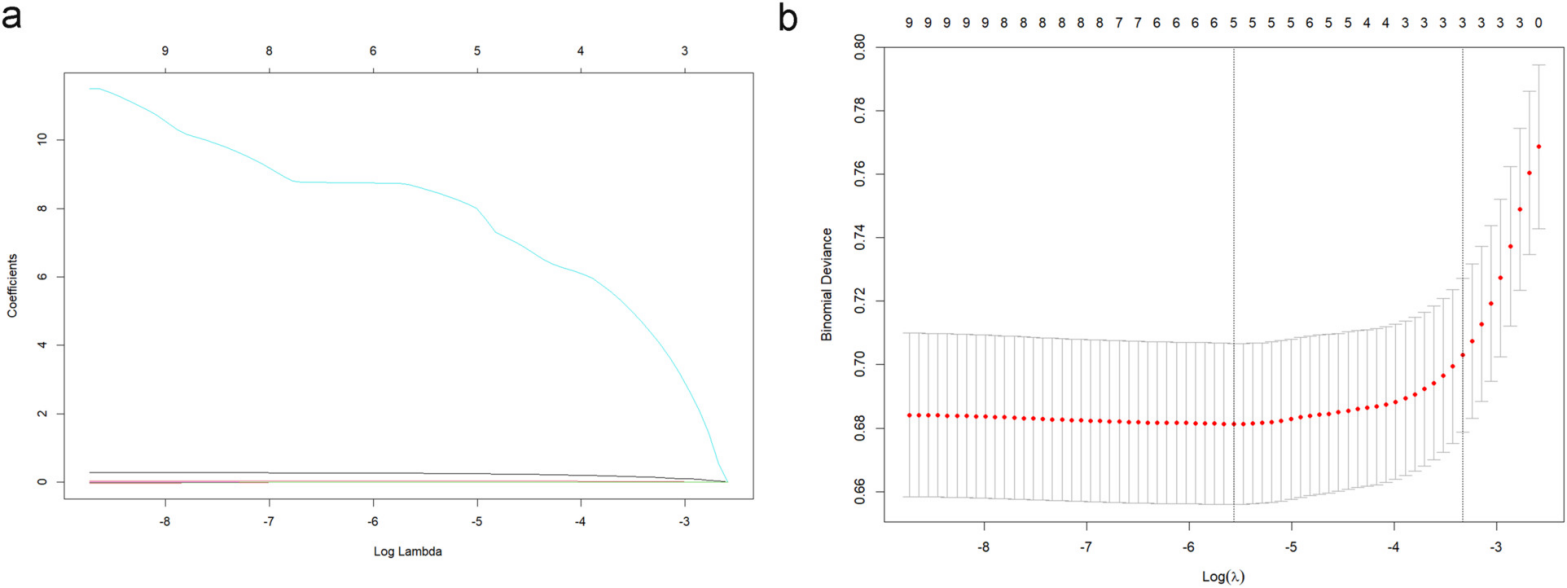
LASSO regression analysis **(a)** and cross-validation visualization results **(b)**.

**Figure 5:**
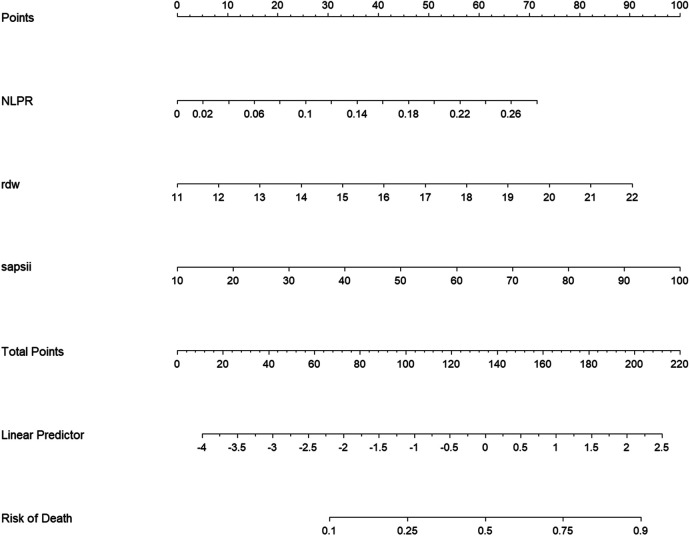
Column line diagram of the new model. NLPR, neutrophil-lymphocyte platelet ratio; rdw, erythrocyte distribution width; SAPSII, simplified acute physiology score II.

Based on the aforementioned results, it was proposed that NLR, NLPR, RDW, and SIRI can serve as independent predictive factors for the likelihood of long-term mortality of the NSTEMI population in the ICU. These factors could be integrated with current widely-used clinical prognostic scoring systems to construct a novel predictive model for further assessing the long-term prognosis in individuals with severe NSTEMI. Data were extracted from the MIMIC on 1,607 patients and the cohort was split into training (*N* = 1,125) and validation (*N* = 482) sets at a 7:3 ratio. Variables with predictive value were encompassed in a LASSO regression analysis to identify those with the most significant impact on outcomes, which were incorporated into the final predictive model. To determine the optimal penalty coefficient (*λ*) for defining the variables to be included in the new model, cross-validation was conducted. The results of LASSO regression and cross-validation visualizations are shown in Figure 4. The final *λ* value was determined to be *λ*1se = 0.043. NLPR, RDW, and SAPII scores were utilized as modeling variables for model construction. Finally, the model variables were entered into a logistic regression model to create a nomogram (Figure 5).

### Validation of the predictive model

**Figure 6:**
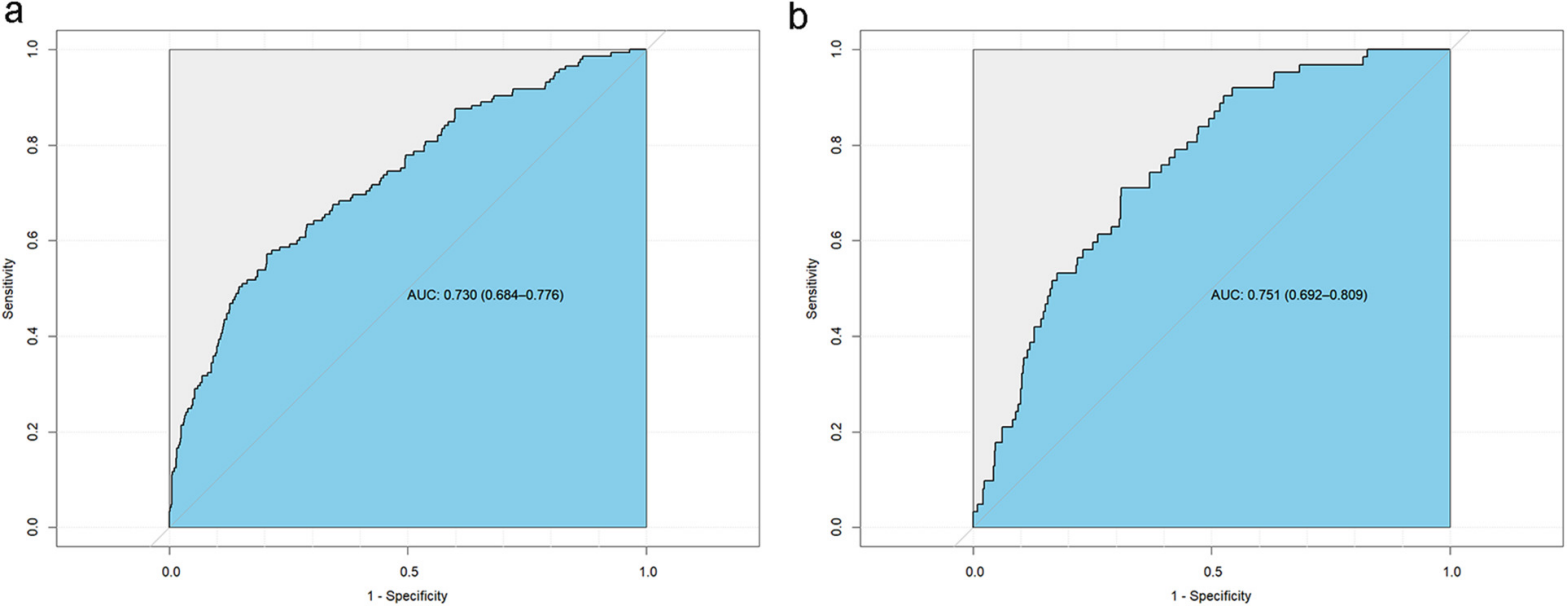
Subject operating curves (ROC) for the new model. **(a)** Training set ROC curve, **(b)** Validation set ROC curve.

**Figure 7:**
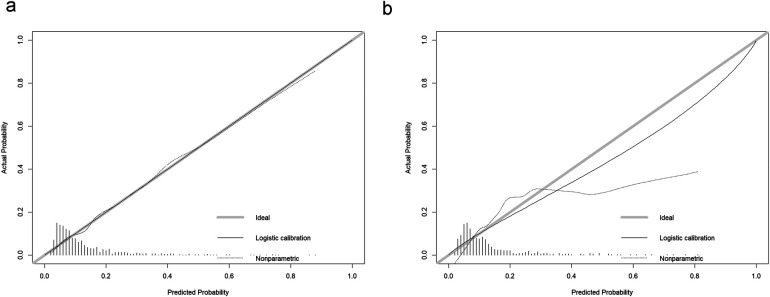
Calibration curves for the new model. **(a)** Training set calibration curve, **(b)** Validation set calibration curve.

The model's predictive performance was subsequently validated in both sets. The ROC curve for the model demonstrated a sensitivity of 0.57, a specificity of 0.79, and an AUC of 0.730 (95% CI: 0.684–0.776) in the training set, and a sensitivity of 0.71, a specificity of 0.69, and an AUC of 0.751 (95% CI: 0.692–0.809) in the validation set, as shown in Figure 6. Furthermore, calibration curve analysis indicated that while the predicted curve in the training set slightly deviated from the ideal reference curve, there was still good agreement between observed and predicted outcomes (Figure 7).

## Discussion

Previous studies have extensively explored the potential value of various inflammatory markers in the diagnosis and prognostic prediction of cardiovascular diseases. Among these, NLR has demonstrated strong utility in assessing both the severity and prognosis of CHD. For instance, S. Yuan et al. reported a significant association between NLR and higher Gensini scores, indicating its predictive value in evaluating the extent of coronary artery lesions in patients with acute coronary syndrome (ACS) (5). Similarly, PLR has been found to assist in risk stratification for adverse events in CHD. J. Larmann et al. observed that elevated PLR was associated with perioperative cardiovascular complications in patients with CHD (6). In addition, a meta-analysis conducted by C. Zhang et al. demonstrated that SII was independently associated with an increased risk of major adverse cardiovascular events (MACE) following percutaneous coronary intervention (PCI) in CHD patients (7). Moreover, T. Y. Zhang found that individuals with CHD exhibited higher SIRI levels (8).Y. Jiang's study revealed that the aggregate index of systemic inflammation (AISI) was an effective predictor of short-term adverse outcomes in patients with acute myocardial infarction, and was also closely associated with the incidence of new-onset atrial fibrillation and contrast-induced nephropathy (9). Other studies have identified an independent association between NLPR and poor short-term prognosis in CHD patients admitted to the ICU, suggesting a potential prognostic value of NLPR in critical care settings (10). Additionally, elevated RDW is independently and incrementally associated with mortality risk among patients with CHD, a relationship that may reflect chronic or abnormal inflammatory conditions (11). Y. Cheng et al. integrated these inflammatory indices to further investigate their association with in-hospital and short-term mortality risks in ICU patients with CHD. Their findings indicated that all aforementioned inflammatory markers were independent predictors of short-term mortality in this population (10).

This study incorporated a variety of inflammatory markers to compare and clarify their roles in risk assessment for patients with severe NSTEMI. It was found that inflammatory markers (NLR, NLPR, RDW, SIRI) were significantly associated with long-term (180-day and 360-day) risk of death in ICU individuals suffering severe NSTEMI. After adjusting for multiple confounding factors, these associations persisted. Moreover, a positive relation was proved between higher levels of composite inflammatory markers and an increased risk of mortality, suggesting the potential value of these markers in the long-term risk management of NSTEMI. Subsequent sensitivity analyses confirmed that NLR, RDW, and NLPR were independent predictors of long-term mortality in ICU patients with NSTEMI, unaffected by coexisting malignancies or autoimmune disorders. Notably, after adjusting for potential confounders, the hazard ratios of these indices increased, underscoring their clinical utility. In contrast, the predictive value of SII and SIRI may be dependent on the presence of chronic inflammatory states and should be interpreted with caution. These results reinforce the robustness of the primary analyses and provide a rationale for prioritizing NLR, RDW, and NLPR as prognostic biomarkers in clinical practice. Finally, the study further developed a novel predictive model that may provide more viable options for future clinical decision-making and risk management.

Increased NLR, NLPR, RDW, and SIRI levels correlated with poor prognosis in ICU patients with NSTEMI, which was consistent with the conclusions of many prior studies. Among these, NLR is a traditional and widely applied composite inflammatory marker, which to some extent reflects the balance between systemic inflammatory response and immune response. Neutrophils, as part of this response, indicate the acute inflammation process in patients and may also contribute to the advanced stages of CAD via inducing smooth muscle dissolution and death in atherosclerotic plaques, thereby destabilizing the plaque (12). Lymphocytes are also important in regulating atherosclerosis. More Th1 cells are expressed at sites of atherosclerotic lesions, which, via interaction with macrophages and endothelial cells, produce pro-inflammatory factors that further promote the progression of atherosclerosis, while regulatory T cells (Tregs) have a protective role in this process (13). A substantial body of research has identified the prognostic value of NLR in cardiovascular diseases, particularly in ACS. A meta-analysis demonstrated that NLR is related to both short-term and long-term death in the NSTEMI population and serves as a crucial indicator for predicting adverse cardiovascular outcomes (14). RDW, traditionally used to assess anemia, is an indicator of red blood cell size heterogeneity. Recent research has revealed that RDW influences chronic inflammation and oxidative stress and is an independent predictor of poor prognosis in CAD sufferers. A study by T.T. Wu et al. found that high levels of RDW are an independent risk factor for cardiogenic death in CAD patients undergoing PCI; when RDW ≥ 13.1%, the incidence of cardiogenic death increased by 1.33 times (15). As another marker of the inflammatory response, SIRI has been recognized as a key indicator of vascular inflammation and lesion severity in CAD patients. This index integrates NEUT, MONO and LC to show the multi-layered inflammatory response in the body. J. Guo et al. found that, compared to NLR, SIRI, an independent risk factor for STEMI, showed a stronger correlation with the Gensini score, thus aiding in the assessment of coronary lesion severity (16). Additionally, NLPR, which combines changes in neutrophils, platelets, and lymphocytes, offers a more comprehensive reflection of the inflammatory burden. The inclusion of platelet count further enhances its ability to reflect coagulation function and inflammatory status. In our study, the elevation of NLPR was significantly linked to long-term death of patients, which aligns with the findings of Cheng et al., whose research showed that NLPR is a strong predictor of short-term death of CHD individuals in the ICU (10). However, our study revealed a relative scarcity of research on the association between NLPR and CHD risk, indicating the need for further studies to explore the clinical value of NLPR in CAD.

It is noteworthy that in this study, no independent association was found between PLR and ACM at 180 and 360 days in ICU patients with NSTEMI. In comparison to other composite inflammatory markers (NLR, SIRI, and RDW), the role of PLR in forecasting the likelihood of long-term death is relatively weak, which contradicts the findings of many previous studies. It was hypothesized that the reasons for this discrepancy were possibly attributed to the following factors: (1). The dynamic changes in lymphocyte levels may weaken the stability of PLR. The calculation of PLR relies on lymphocyte count, which may be influenced by various factors, including chronic stress, aging, and medication use (such as corticosteroids) (17, 18). These factors may cause fluctuations in PLR during the chronic phase following NSTEMI, thereby reducing its stability and sensitivity as a long-term prognostic marker. Additionally, in ICU patients with NSTEMI, lymphocyte levels may be persistently suppressed due to severe infections (19) and multi-system complications (20), while platelet levels may remain relatively stable. This imbalance could result in PLR being unable to accurately reflect the long-term inflammatory burden and immune function of the patients. (2). The functional diversity of platelets may dilute the predictive role of PLR. Platelets play diverse roles in thrombosis, inflammation regulation, and immune response (21). However, the calculation of PLR is solely based on platelet count, which does not capture dynamic changes in platelet function. For example, activated platelets release various pro-coagulant, pro-inflammatory, and vasoactive mediators, and they can participate in plaque progression through upregulating the release of pro-inflammatory factors like IL-1β, IL-8, tumor necrosis factor (TNF)-α, as well as adhesion molecules (22). These functional changes may be unrelated to simple platelet count. Therefore, the use of PLR alone may be insufficient to reflect the diverse functions of platelets in different pathophysiological states. Studies have also shown that the effectiveness of PLR may be influenced by other markers. For instance, a combined model of NLR and PLR demonstrates a higher predictive ability for short-term AEs in CAD patients, while the predictive ability of PLR alone is limited (23). (3). The clinical heterogeneity of PLR may contribute to inconsistent results. The applicability of PLR may be influenced by the heterogeneity of the patient population. For example, chronic illnesses like diabetes, chronic kidney disease, and malignancy may significantly elevate baseline PLR values, thus masking its specific reflection of the inflammatory state. Plenty of studies have demonstrated significantly higher PLR levels in diabetic or CAD patients with diabetes than in non-diabetic patients (24, 25), which may interfere with the predictive ability of PLR in these patients. Owing to the limitations of the database, it was impossible to fully adjust for all comorbidities that may affect PLR, which may have impacted the results. (4). The statistical methods employed for PLR in this study may limit its predictive ability. The independent association of PLR was assessed using the Cox regression model. Although several covariates were adjusted for, there may still be uncontrolled confounding factors. For example, certain clinical features not included in the model (such as chronic inflammatory burden or metabolic state) may have interfered with the independent predictive ability of PLR.

AMI is mainly caused by the rupture of atherosclerotic plaques in the coronary arteries, resulting in the formation of acute thrombosis and coronary vasospasm, which subsequently results in acute occlusion of the coronary lumen and severe myocardial ischemic necrosis. Inflammation is an integral process throughout the entire pathogenesis and progression of the disease. First, various inflammatory cells and mediators influence the erosion of unstable plaques and trigger plaque rupture. Macrophages aggregated within the plaque release matrix metalloproteinases (MMPs) and other enzymes to degrade extracellular components, such as collagen, thereby weakening the mechanical strength of the fibrous cap (26). Furthermore, the inflammatory microenvironment, consisting of interferon (INF)-γ, macrophage colony-stimulating factor (M-CSF), monocyte chemoattractant protein (MCP)-1, and IL-1, enhances macrophage activity, accelerates fibrous cap damage, and induces endothelial inflammatory activation, thereby recruiting additional pro-inflammatory cells (27). During the acute phase of myocardial infarction, while the inflammatory response facilitates tissue repair, it also exacerbates myocardial injury. Neutrophils, via the release of reactive oxygen species (ROS) and various proteases, clear necrotic myocardial tissue. However, excessive neutrophil activity can damage still-surviving myocardial cells, thus expanding the area of myocardial injury (28). In addition, neutrophils form extracellular traps (NETs), which further exacerbate local inflammation and thrombus formation (29). Monocytes in the acute phase of myocardial infarction differentiate into pro-inflammatory M1 macrophages, which secrete IL-6 and TNF-α, further activating local inflammatory pathways. The persistent activation of the inflammatory response also intensifies myocardial injury and ventricular remodeling (30, 31). Classic inflammatory signaling pathways, including Toll-like receptors (TLRs)-nuclear factor κB (NF-κB), are significantly activated during myocardial ischemia-reperfusion injury. These signals not only regulate the expression of pro-inflammatory factors but also promote adverse post-infarction events through inflammatory cell recruitment and platelet activation (32, 33). Inflammation also plays a role in long-term repair following myocardial infarction. During the repair phase after infarction, monocytes gradually transition from M1 macrophages to repair-type M2 macrophages, contributing to tissue repair. Delayed conversion of this macrophage population may result in incomplete scar formation, further increasing the risk of long-term heart failure (34, 35). Additionally, patients suffering NSTEMI may experience prolonged chronic inflammation, manifested by persistently elevated inflammatory markers such as CRP, which may be associated with adverse long-term outcomes, including worsening heart function and recurrent cardiovascular events. By exploring the mechanisms of inflammatory response in AMI, this research can further assist clinicians in making better treatment decisions for NSTEMI patients and provide potential new therapeutic targets for anti-inflammatory treatment in myocardial infarction.

Our study offers a novel perspective on the management of long-term prognosis in the acute NSTEMI population in the ICU. Prior research mainly focused on the assessment of short-term prognosis in ACS patients based on inflammatory markers. However, studies specifically addressing the long-term prognosis of NSTEMI patients, particularly those with severe conditions requiring ICU admission for further treatment, are relatively scarce. This study first examined the association of inflammatory markers with long-term prognosis in ICU patients suffering from NSTEMI, highlighting the impact of their inflammatory burden on long-term outcomes. Furthermore, our study systematically analyzes a range of inflammatory markers, incorporating both multi-factorial indicators (like NLR, PLR, and SII) and single inflammatory markers (such as RDW), thereby providing clinicians with a more comprehensive perspective. Novel inflammatory markers, such as NLPR and AISI, were also included for evaluating long-term prognosis in NSTEMI individuals, thus offering additional possibilities for inflammatory evaluation in this cohort. Through comparisons of the predictive efficacy of various inflammatory markers, potential limitations of PLR in forecasting long-term prognosis in people suffering severe NSTEMI were identified, and the underlying biological mechanisms that may explain this phenomenon were explored, providing valuable insights for future research. In the study, a new predictive model was developed by integrating variables such as NLPR, RDW, and SAPII scores, and this model was optimized using LASSO regression and nomograms. Compared to the traditional SAPII score, the new model, which integrates inflammatory markers, offers a better reflection of the patient's inflammatory burden. Additionally, the data were sourced from the internationally recognized MIMIC-IV ICU database, which features a large sample size, high data integrity, and coverage of various clinical variables. Stringent inclusion/exclusion criteria and multiple imputation methods ensured the scientific rigor and statistical robustness of the study's conclusions.

However, our study has limitations. First, the data are from the MIMIC-IV, involving ICU patients from a single medical center in Massachusetts, USA, with a predominantly Caucasian population. This single-center data source may overlook regional and racial differences that could influence the results, thus limiting the generalizability of the study's conclusions, particularly regarding the impact of racial differences on CAD prognosis. Additionally, the inflammatory markers used in this study were based on a single-point measurement at ICU admission, and such static data may not fully capture the inflammatory response process over the course of the patient's illness. Furthermore, the inflammatory burden in ICU patients may fluctuate due to various factors, including anti-inflammatory medications, hospital-acquired infections, and infection control measures. Single-point measurements may also obscure the true significance of certain markers during specific time windows, such as during inflammatory flare-ups or stabilization. Finally, as a retrospective study, the completeness and accuracy of the data are limited by the quality of the database, which may introduce potential biases. Moreover, retrospective studies are unable to establish causality, meaning that it remains unclear whether a higher inflammatory burden is the cause of long-term adverse prognosis or a consequence of severe disease. To clarify the causal relationship, further prospective cohort studies are needed.

In the future, multi-center prospective cohort studies are necessitated to diversify sample sources and increase the diversity of the samples, thereby enhancing the generalizability of the model. At the same time, it is crucial to ensure the clarity of causal relationships and further verify the forecasting value of inflammatory markers in the real world. Moreover, long-term follow-up studies are necessary to capture the dynamic changes of inflammatory markers through time-series analysis, evaluating the impact of their temporal effects on long-term prognosis.

## Conclusion

This study identified significant associations between NLR, NLPR, RDW, and SIRI with long-term mortality in ICU patients diagnosed with NSTEMI. A novel prognostic model integrating NLPR and RDW with SAPS II was developed, offering a potentially effective tool for risk stratification and clinical decision-making in critically ill NSTEMI patients.

## Data Availability

The original contributions presented in the study are included in the article/Supplementary Material, further inquiries can be directed to the corresponding author.
